# Airway Complications during and after General Anesthesia: A Comparison, Systematic Review and Meta-Analysis of Using Flexible Laryngeal Mask Airways and Endotracheal Tubes

**DOI:** 10.1371/journal.pone.0158137

**Published:** 2016-07-14

**Authors:** Rui Xu, Ying Lian, Wen Xian Li

**Affiliations:** 1 Department of Anesthesiology, the Eye, Ear, Nose and Throat Hospital of Fudan University, Shanghai Medical College of Fudan University, Shanghai, China; 2 Department of Case Administration, Shandong Provincial Qian Foshan Hospital of Shandong University, Jinan, China; University of Pennsylvania, UNITED STATES

## Abstract

**Objective:**

Flexible laryngeal mask airways (FLMAs) have been widely used in thyroidectomy as well as cleft palate, nasal, upper chest, head and neck oncoplastic surgeries. This systematic review aims to compare the incidence of airway complications that occur during and after general anesthesia when using the FLMA and endotracheal intubation (ETT). We performed a quantitative meta-analysis of the results of randomized trials.

**Methods:**

A comprehensive search of the PubMed, Embase and Cochrane Library databases was conducted using the key words "flexible laryngeal mask airway" and "endotracheal intubation". Only prospective randomized controlled trials (RCTs) that compared the FLMA and ETT were included. The relative risks (RRs) and the corresponding 95% confidence intervals (95% CIs) were calculated using a quality effects model in MetaXL 1.3 software to analyze the outcome data.

**Results:**

Ten RCTs were included in this meta-analysis. There were no significant differences between the FLMA and ETT groups in the incidence of difficulty in positioning the airway [RR = 1.75, 95% CI = (0.70–4.40)]; the occurrence of sore throat at one hour and 24 hours postoperative [RR = 0.90, 95% CI = (0.13–6.18) and RR = 0.95, 95% CI = (0.81–1.13), respectively]; laryngospasms [RR = 0.58, 95% CI = (0.27–1.23)]; airway displacement [RR = 2.88, 95% CI = (0.58–14.33)]; aspiration [RR = 0.76, 95% CI = (0.06–8.88)]; or laryngotracheal soiling [RR = 0.34, 95% CI = (0.10–1.06)]. Patients treated with the FLMA had a lower incidence of hoarseness [RR = 0.31, 95% CI = (0.15–0.62)]; coughing [RR = 0.28, 95% CI = (0.15–0.51)] during recovery in the postanesthesia care unit (PACU); and oxygen desaturation [RR = 0.43, 95% CI = (0.26–0.72)] than did patients treated with ETT. However, the incidence of partial upper airway obstruction in FLMA patients was significantly greater than it was for ETT patients [RR = 4.01, 95% CI = (1.44–11.18)].

**Conclusion:**

This systematic review showed that the FLMA has some advantages over ETT because it results in a lower incidence of hoarseness, coughing and oxygen desaturation. There were no statistically significant differences in the difficulty of intubation or in the occurrence of laryngospasms, postoperative sore throat, airway displacement, aspiration or laryngotracheal soiling. However, there was a higher incidence of partial upper airway obstruction in the FLMA than in the ETT group. We conclude that the FLMA has some advantages over ETT, but surgeons and anesthesiologists should be cautious when applying the mouth gag, moving the head and neck, or performing oropharyngeal procedures to avoid partial upper airway obstruction and airway displacement. The FLMA should not be used on patients at high risk for aspiration.

## Introduction

The laryngeal mask airway (LMA) was developed by Dr. Brain in 1981, and since then, it has flourished in practice and been used to treat millions of patients worldwide. The LMA provides more hands-free anesthesia than a facemask does, avoids many morbidities associated with tracheal intubation because there is no stress from the laryngoscope, and allows a faster recovery that does not require muscle relaxation [1,2]. The LMA has become an important choice for routine use, particularly in outpatient surgeries [3]. It has been recommended that all hospitals have LMAs available for unanticipated rescue intubations or intubations classified as difficult by the Difficult Airway Society 2015 guidelines and the 4th National Audit Project of the Royal College of Anaesthetists and Difficult Airway Society (NAP4) [4].

Various types of LMAs have been developed. The flexible laryngeal mask airway (FLMA) was first used successfully in tonsillectomies and dental surgeries in 1990 to prevent the obstruction and kinking observed when using classical LMA tubes [5]. Since then, the FLMA has been used in thyroidectomies; cleft palate surgeries; nasal surgeries; and upper chest, head and neck oncoplastic surgeries.

The effectiveness and safety of the FLMA and endotracheal intubation (ETT) have been compared in some randomized controlled trials (RCTs). A lower incidence of some postoperative airway complications has been reported when using the FLMA. In addition, the FLMA has been found to have some shortcomings, such as difficulty of insertion, the possibility of dislocation during the procedure, the risk of ventilation into the esophagus and stomach, and an increased risk of aspiration and partial upper airway obstruction [6,7]. However, many of these studies included small numbers of subjects, and their conclusions with regard to some issues are controversial.

In this study, we sought to determine whether the incidence of airway complications during and after general anesthesia can be reduced by using the FLMA rather than ETT.

## Methods

### Search strategy

Two authors (RX and YL) independently searched the PubMed, Cochrane Library and Embase databases for relevant articles written in English and published from the inception of each database through July 2015. Differences were resolved through discussion. The search terms included “flexible reinforced laryngeal mask airway”, “flexible LMA”, “flexible laryngeal mask”, “FLMA”, “LMA-flexible”, “RLMA”, “reinforced laryngeal mask airway”, “intratracheal intubation”, “intratracheal intubations” “endotracheal intubation”, “endotracheal intubations” and “ETT”. The titles and abstracts of the potentially relevant articles were scanned by the same two authors. The reference lists of all included studies were manually reviewed, and relevant review articles were used to identify potentially eligible articles.

### Eligibility criteria

The following inclusion criterion was used: prospective RCTs containing at least two independent groups that compared the use of the FLMA and ETT in patients who underwent surgery.

All selected articles provided sufficient information to pool the data for the incidence of airway complications using the FLMA and ETT. The evaluated outcomes included difficult intubation, sore throat, hoarse voice, coughing, laryngospasms, oxygen desaturation, aspiration, laryngotracheal soiling, airway displacement and partial upper airway obstruction.

### Exclusion criteria

Any studies that were presented as comments, case reports, crossover studies, letters, editorials or retrospective studies were excluded. We also excluded studies that did not report the outcomes of interest. Comparative studies that used manikins, studies that included tracheostomy procedures or investigated introducer devices, and any other non-relevant studies were excluded.

### Quality assessment and data analysis

The same two authors (RX, YL) independently read the full text articles and determined whether each article was eligible for inclusion in the current meta-analysis. Any disagreements between the authors were resolved through discussion with the corresponding author (WXL).

Quality assessments and scoring of all articles were independently performed by two authors (RX, YL) according to the guidelines of the editorial board of the Cochrane Collaboration Back Review Group (BRG) [8]. If these assessments were inconsistent, the issue was resolved through discussion with the corresponding author (WXL). The quality scoring criteria are shown in Table 1, and the total score of the included studies was eleven.

**Table 1:** Quality scoring system and quality index (Qi).

	Criteria	Score
1. Was the method of randomization adequate?	No	0
	Unclear	0.5
	Yes	1
2. Was the treatment allocation concealed?	No	0
	Unclear	0.5
	Yes	1
3. Was the baseline similar in both groups in terms of the most important prognostic indicators?	No	0
	Unclear	0.5
	Yes	1
4. Were the patients blinded to the intervention?	No	0
	Unclear	0.5
	Yes	1
5. Were the care providers blinded to the intervention?	No	0
	Unclear	0.5
	Yes	1
6. Were the co-interventions similar in both groups?	No	0
	Unclear	0.5
	Yes	1
7. Was the compliance acceptable in both groups?	No	0
	Unclear	0.5
	Yes	1
8. Was the drop-out rate described and acceptable?	No	0
	Unclear	0.5
	Yes	1
9. Was the timing of the outcome assessment in both groups similar?	No	0
	Unclear	0.5
	Yes	1
10. Did the analysis use intention-to-treat where applicable?	No	0
	Unclear	0.5
	Yes	1
Total scores of all studies		11
Quality index (QI) = Sum of the above scores/11		

### Data collection process

The data extraction from each article was performed by two independent authors (RX, YL), including an anesthesiologist and a statistician. The authors were blinded to the title, authors, and journal of each article. The following items were collected from each article: name of the first author; year of publication; country; sample size; patient characteristics; type of surgery and ventilation; American Society of Anesthesiologists (ASA) grade; pre-anesthesia medication; cuff pressure; insertion technique; difficult intubation; and airway complications during and after general anesthesia (sore throat, hoarse voice, coughing, laryngospasms, oxygen desaturation, aspiration, laryngotracheal soiling, airway displacement and partial upper airway obstruction). Discrepancies were resolved through discussion with the corresponding author (WXL).

### Statistical analysis

This meta-analysis of the available RCTs was conducted following the Systematic Reviews and Meta-Analyses (PRISMA) Statement guidelines (see S1 Table) [9] and the recommendations of the Cochrane Collaboration. To explicitly address study heterogeneity caused by differences in the qualities of studies and to produce more definitive results, MetaXL uses a process called the quality effects model to pool outcome data. This model is a modified version of the fixed-effects inverse variance method and gives greater weight to high-quality studies [10,11]. The quality index (QI) of each article was equal to the quality score of that article divided by the total quality score. A quality effect model of MetaXL version 1.3 was then applied to analyze the data and calculate the total relative risk (RR) and 95% confidence interval (95% CI) for dichotomous outcomes. The heterogeneity assumption was assessed by a Chi-square-based Q-test and an I-squared test. If the P value for the Q-test was <0.10, the heterogeneity was significant. Statistical significance was considered if the 95% CIs did not include the value of “1.0” for RR. Publication bias was not assessed due to the limited number of studies included for each individual outcome.

## Results

### Search results

**Fig 1:**
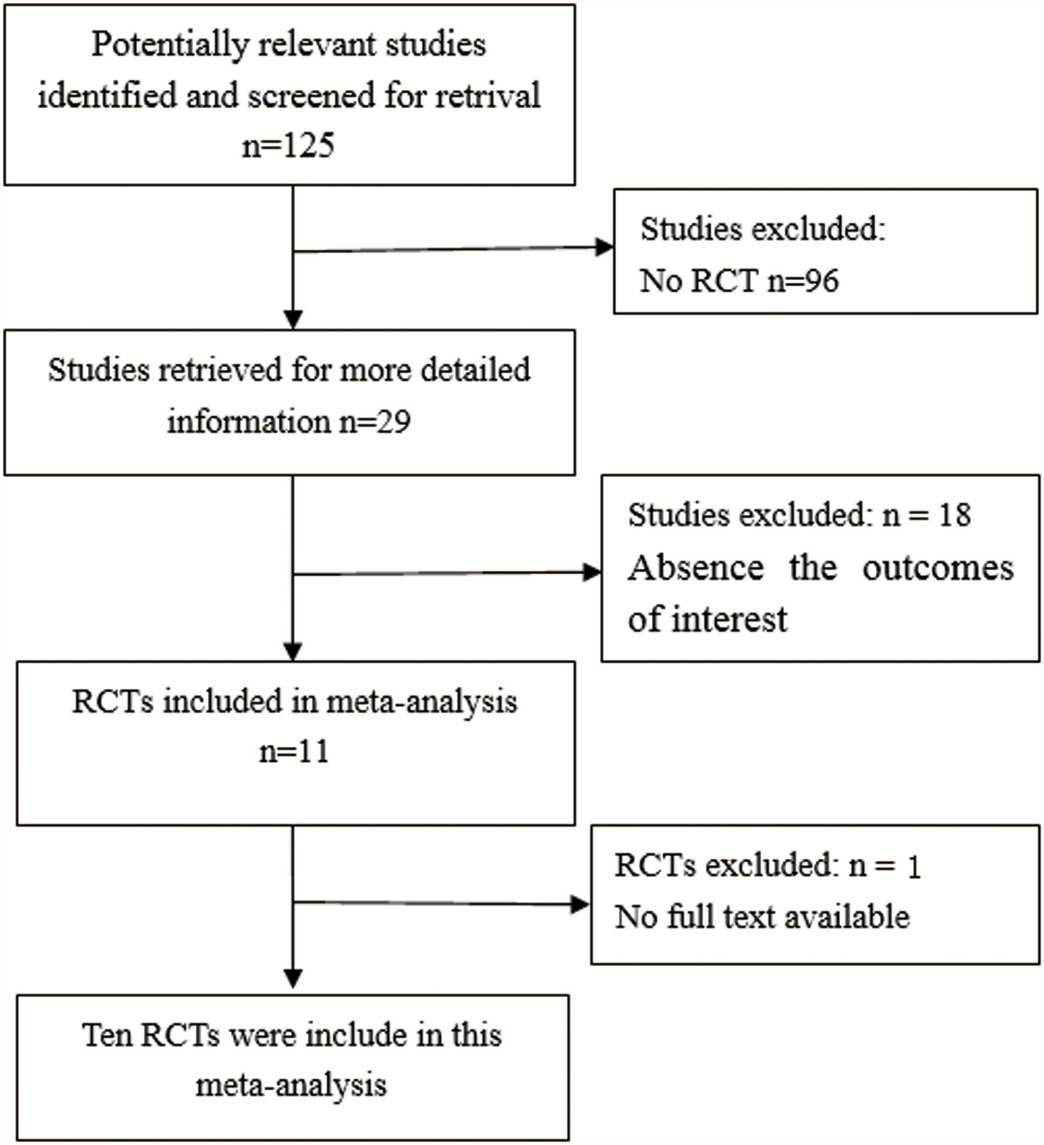
Flow diagram of the process of literature identification, screening, and selection.

A total of 125 records were retrieved for screening through the literature search. Based on the titles, abstracts, and full texts of the articles, 115 records were removed. Finally, ten RCTs involving 996 participants were included in this meta-analysis [6,7,12–19]. The processes for study identification, screening and selection are shown in Fig 1.

### Description of the included trials

The ten studies were British (n = 4), American (n = 1), Norwegian (n = 1), Canadian (n = 1), Indian (n = 1), South Korean (n = 1) and Spanish (n = 1). The 996 participants included 470 from the reinforced laryngeal mask airway (RLMA) group and 536 from the ETT group. All patients underwent elective surgery. Procedures included a thyroidectomy; adenotonsillectomy; nasal surgery; cleft palate surgery; upper chest, head and neck oncoplastic surgery; dentoalveolar surgery; endoscopic intranasal surgery; and septoplasty. The descriptions of the included studies are summarized in Table 2.

**Table 2:** Study characteristics of RCTs comparing FLMA and ETT. (NR means not reported).

Study	Country	Patients (n) (FLMA; ETT)	ASA status	Age (years)	Type of surgery	Cuff pressure (FLMA; ETT)	Insertion technique (FLMA; ETT)	Pre-anesthetic medication	Ventilation (FLMA; ETT)	Quality index
Ryu JH 2013 [12]	South Korea	36; 37	I–II	19–70	Thyroidectomy	(50; 25)	Standard	Midazolam	Mechanical; mechanical	0.95
Doksrød S 2010 [7]	Norway	69; 62	I–II	3–16	Adenotonsillectomy	NR	Guided by the anesthesiologist’s fingers	Midazolam	Mechanical; mechanical	0.91
Peng A 2011 [13]	America	48; 83	I–III	2–12	Adenotonsillectomy	NR	NR	Midazolam hydrochloride	Unknown; unknown	0.77
Kundra P 2009 [14]	India	33; 33	I–II	2–3	Cleft palate surgery	NR	The lateral, partially inflated technique	Midazolam and atropine	Mechanical; mechanical	0.82
Martin-Castro C 2007 [15]	Spain	60; 60	I–III	Above 18	Upper chest, head and neck oncoplastic surgery	(60; 30)	Standard	NR	Mechanical; mechanical	0.86
Webster AC 1999 [6]	Canada	35; 66	I–II	Above 18	Endoscopic intranasal surgery or septoplasty	NR	Guided by the anesthesiologist’s fingers	Gallamine	Spontaneous; mechanical/ spontaneous	0.77
Williams P 1993 [16]	British	48; 52	I–II	3–37	Adenotonsillectomy	NR	NR	Trimeprazine and tropine (children), papaveretum and hyoscine (adult)	Spontaneous; spontaneous	0.77
Quinn AC 1996 [17]	British	50; 50	I–II	18–60	Dentoalveolar surgery	NR	Standard	NU	Spontaneous; mechanical	0.73
Webster AC 1993 [18]	British	55; 54	I–II	children	Adenotonsillectomy	NR	NR	NU	Spontaneous; spontaneous	0.73
Williams PJ 1995 [19]	British	36; 29	I–II	18–66	Nasal surgery	NR	Standard	Papavereturn and hyoscine	Spontaneous; spontaneous	0.77

Ten outcomes were examined in the ten studies selected for data extraction. The ten outcomes were difficult intubation, sore throat, hoarse voice, coughing, laryngospasms during the postanesthesia care unit (PACU) recovery time, oxygen desaturation, laryngotracheal soiling, airway displacement, aspiration and partial airway obstruction. Difficult intubation was reported in five studies, sore throat in three studies, hoarse voice in two studies, coughing in seven studies, laryngospasms in six studies, oxygen desaturation in nine studies, laryngotracheal soiling in four studies, airway displacement in three studies, aspiration in two studies, and partial airway obstruction in four studies.

### Meta-analysis results

#### Difficult intubation

**Fig 2:**
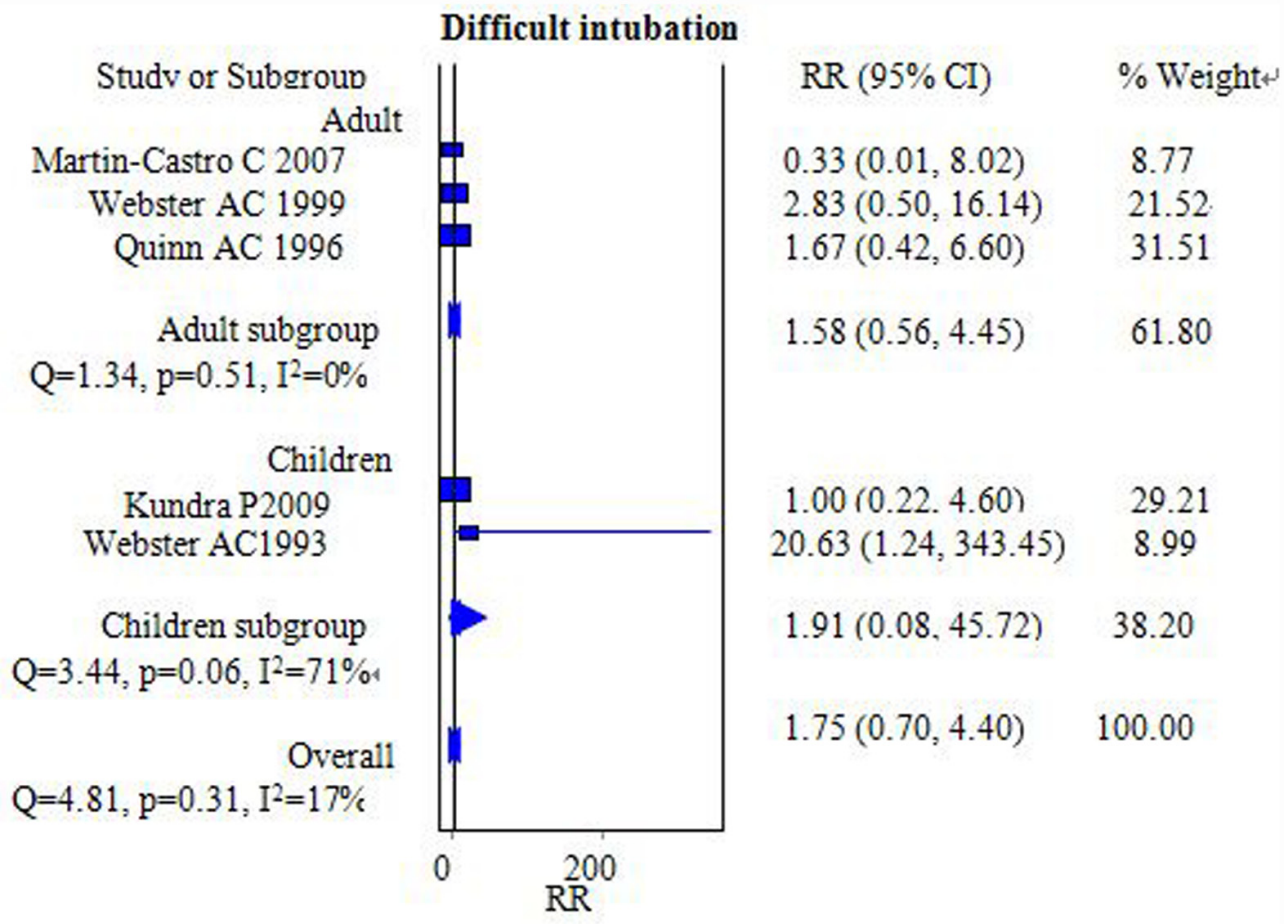
Forest plot demonstrating the incidence of difficulties with positioning the airway. Subgroup analysis based on population age (adults versus children). The incidence was similar between the two groups.

The incidence of difficulties with positioning the airway was investigated in five studies that included 496 participants [6,14,15,17,18]. By pooling the data, it was found that the incidence of difficulties with positioning the airway was similar between the FLMA and ETT groups [RR = 1.75, 95% CI = (0.70–4.40); I^2^ = 17%] (Fig 2). Webster AC et al reported that there was difficulty inserting the FLMA in ten cases compared with none in the ETT group. Sensitivity analyses showed no significant changes in the pooled effect and 95% CI after excluding that study.

Subgroup analyses were performed based on the age of the population, but no further differences were observed in adult [RR = 1.58, 95% CI = (0.56–4.45); I^2^ = 0%] or pediatric [RR = 1.91, 95% CI = (0.08–45.72); *I*^*2*^ = 71%] subgroups (Fig 2).

#### Sore throat

**Fig 3:**
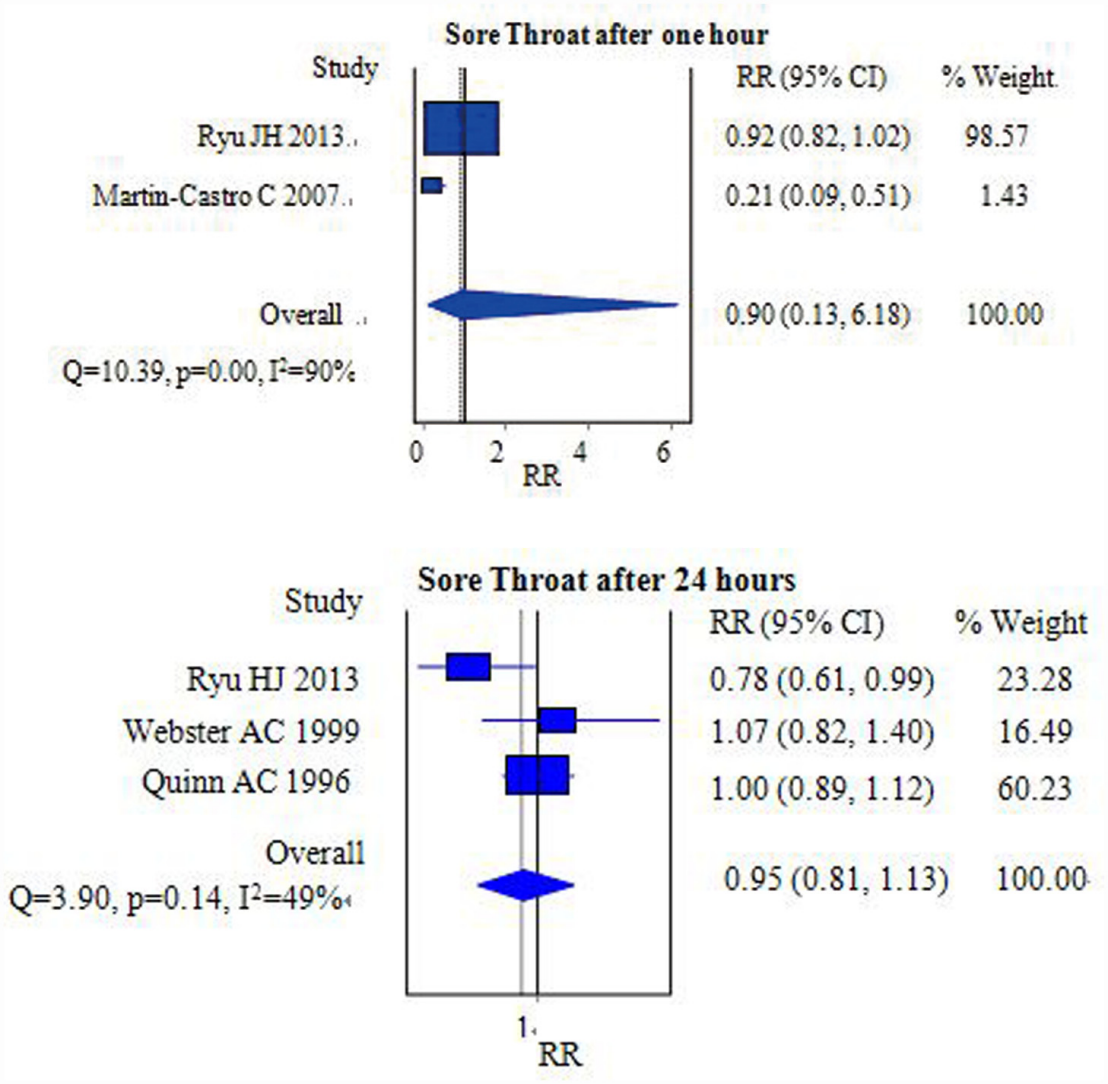
Forest plot demonstrating the incidence of sore throat after one hour (Fig 3a) and the incidence of sore throat after 24 hours (Fig 3b). The incidence was similar between the two groups.

The occurrences of sore throat at one hour and twenty-four hours postoperative were mentioned in two [12,15] and three [6,12,17] studies, respectively. After pooling these data, we found that the incidence of postoperative sore throat was similar between the FLMA [RR = 0.90, 95% CI = (0.13–6.18); I^2^ = 90%] (Fig 3a) and ETT groups [RR = 0.95, 95% CI = (0.81–1.13); *I*^*2*^ = 49%] (Fig 3b).

#### Hoarse voice

**Fig 4:**
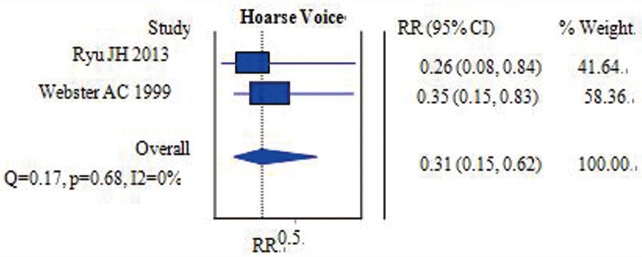
Forest plot demonstrating the incidence of a hoarse voice. The incidence was lower in the FLMA group than in the ETT group.

Two studies with 174 participants compared the incidence of a hoarse voice on the first postoperative day between the two groups [6,12]. Pooled data from these two studies revealed that there was a significant difference between the two groups. Hoarse voice was more common in the ETT group than in the FLMA group [RR = 0.31, 95% CI = (0.15–0.62); I^*2*^ = 0%] (Fig 4) on the first postoperative day. Analysis suggested that the heterogeneity among the trials was not significant.

#### Coughing

**Fig 5:**
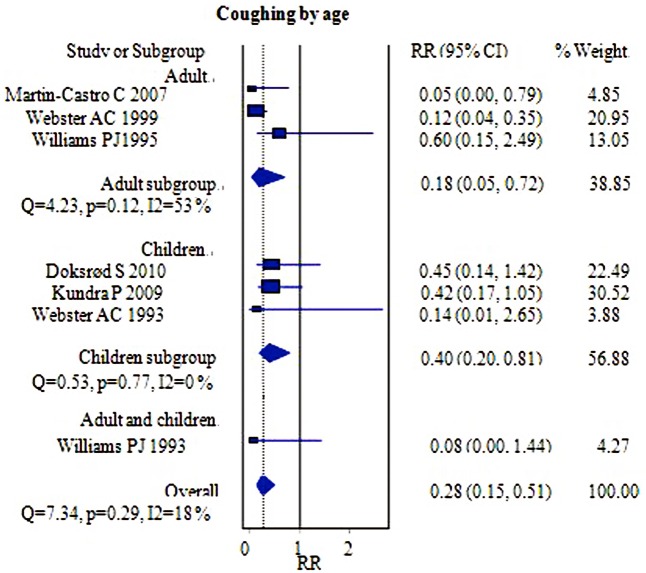
Forest plot demonstrating the incidence of coughing during PACU recovery time. Subgroup analysis according to population age (adults versus children). The incidence was lower in the FLMA group.

Seven studies with 692 patients provided data regarding the numbers of patients experiencing coughing during the PACU recovery time [6,7,14–16,18,19]. The FLMA group had a significantly lower incidence of coughing during the PACU recovery time than did the ETT group [RR = 0.28, 95% CI = (0.15–0.51); I^2^ = 18%] (Fig 5). No heterogeneity was observed. The FLMA group was associated with less coughing during the PACU recovery than was the ETT group in the adult versus children subgroup analysis [RR = 0.18, 95% CI = (0.05–0.72)] and [RR = 0.40, 95% CI = (0.20–0.81)], respectively (Fig 5).

#### Oxygen desaturation

**Fig 6:**
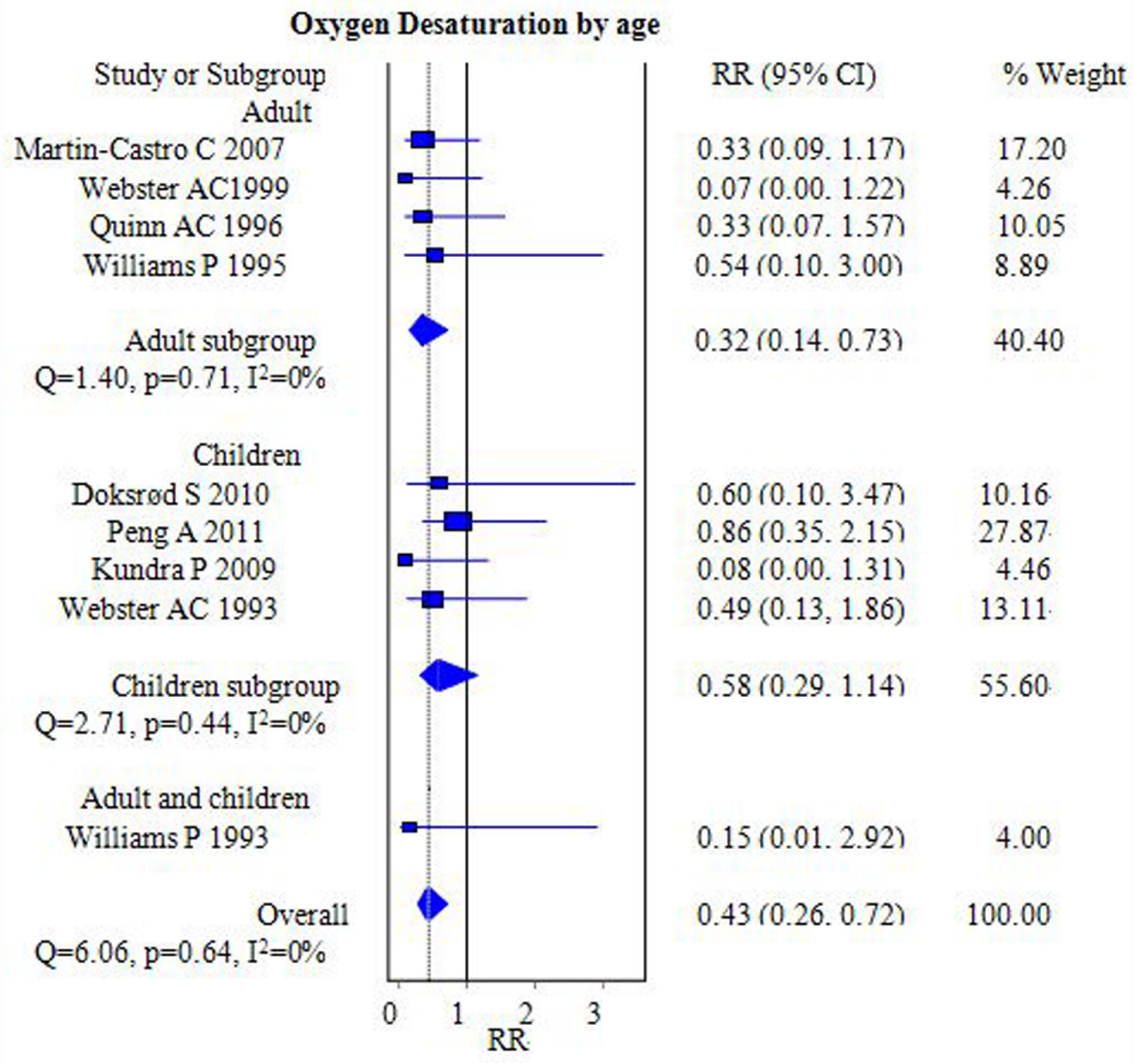
Forest plot demonstrating the incidence of postoperative oxygen desaturation. Subgroup analysis according to population age (adults versus children). The incidence was lower in the FLMA group.

Nine studies that included 923 participants compared postoperative oxygen desaturation between the FLMA and ETT groups [6,7,13–19]. However, the definition of oxygen desaturation varied among studies, with desaturation defined as SpO_2_ <95% [14,15,17], <94% [16,18,19] and <92% [7]. Some authors did not provide the definition [6,13]. After pooling the incidence of oxygen desaturation, we found that the incidence in the FLMA group was lower than that in the ETT group [RR = 0.43, 95% CI = (0.26–0.72); *I*^*2*^ = 0%] (Fig 6). There was no statistical evidence of heterogeneity in these trials.

The subgroup analysis suggested that oxygen desaturation in the FLMA group was lower than in the ETT group in the adult subgroup [RR = 0.32, 95% CI = (0.14–0.73); *I*^*2*^ = 0%] (Fig 6) but not in the pediatric subgroup [RR = 0.58, 95% CI = (0.29–1.14); *I*^*2*^ = 0%] (Fig 6).

#### Laryngospasms

**Fig 7:**
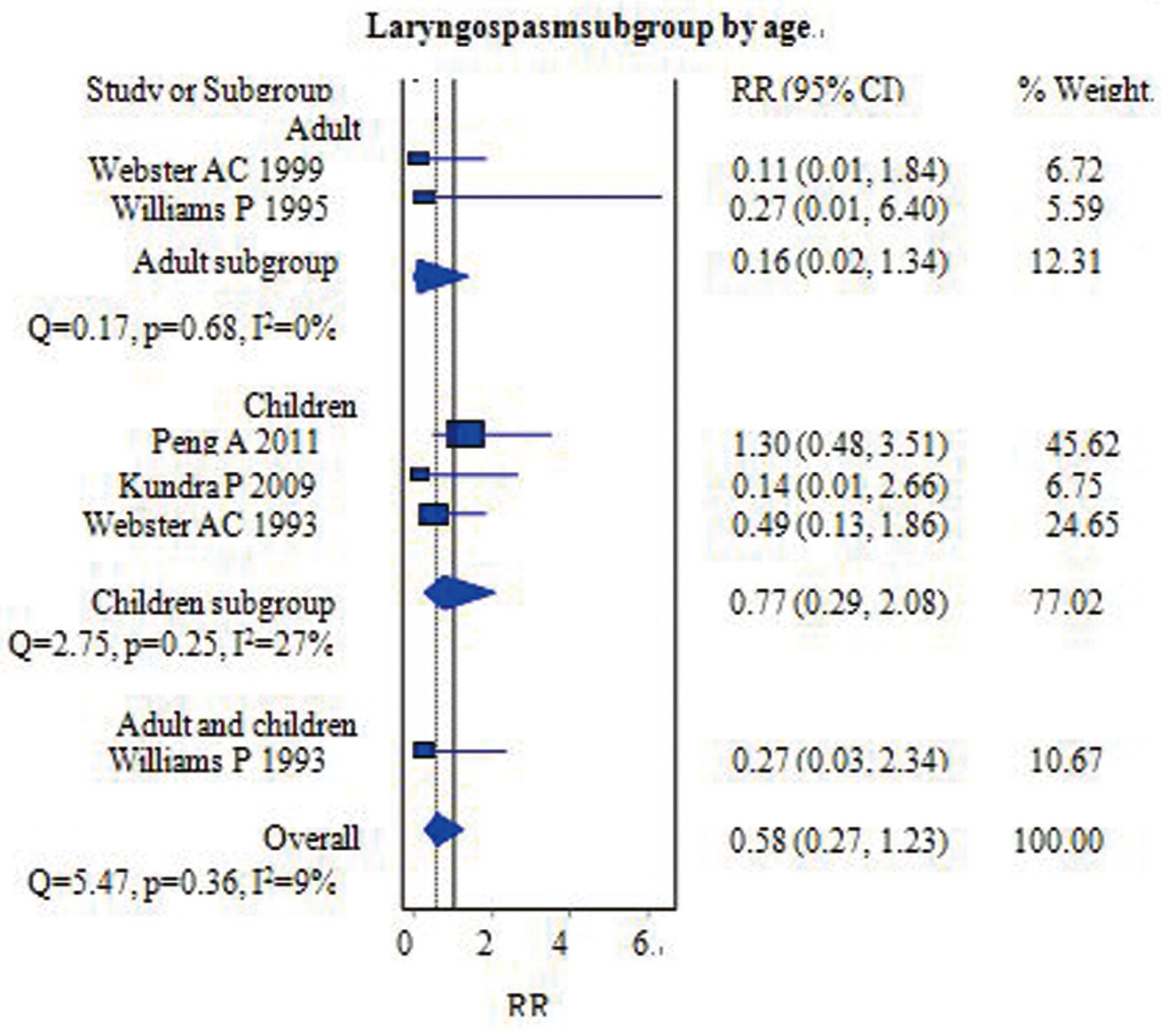
Forest plot demonstrating the incidence of laryngospasms during PACU recovery time. Subgroup analysis according to population age (adults versus children). The incidence was similar between the two groups.

Postoperative laryngospasms were reported in six studies that included 572 patients [6,13,14,16,18,19]. The overall pooled RR of the incidence of laryngospasms following removal of the airway was 0.58 [95% CI = (0.27–1.23); *I*^*2*^ = 9%] (Fig 7). There were no statistically significant differences between the FLMA and ETT groups. Of the six studies, four that included 406 patients compared the incidence of laryngospasms in children, and two (174 patients) compared the incidence in adults. No significant differences were found between the adult and pediatric subgroups after pooling the data [RR = 0.16, 95% CI = (0.02–1.34); *I*^*2*^ = 0%] and [RR = 0.77, 95% CI = (0.29–2.08); *I*^*2*^ = 17%], respectively (Fig 7). Analysis suggested that moderate heterogeneity among the pediatric group influenced the results. No further subgroup analyses were performed due to the limited number of studies.

#### Laryngotracheal soiling

**Fig 8:**
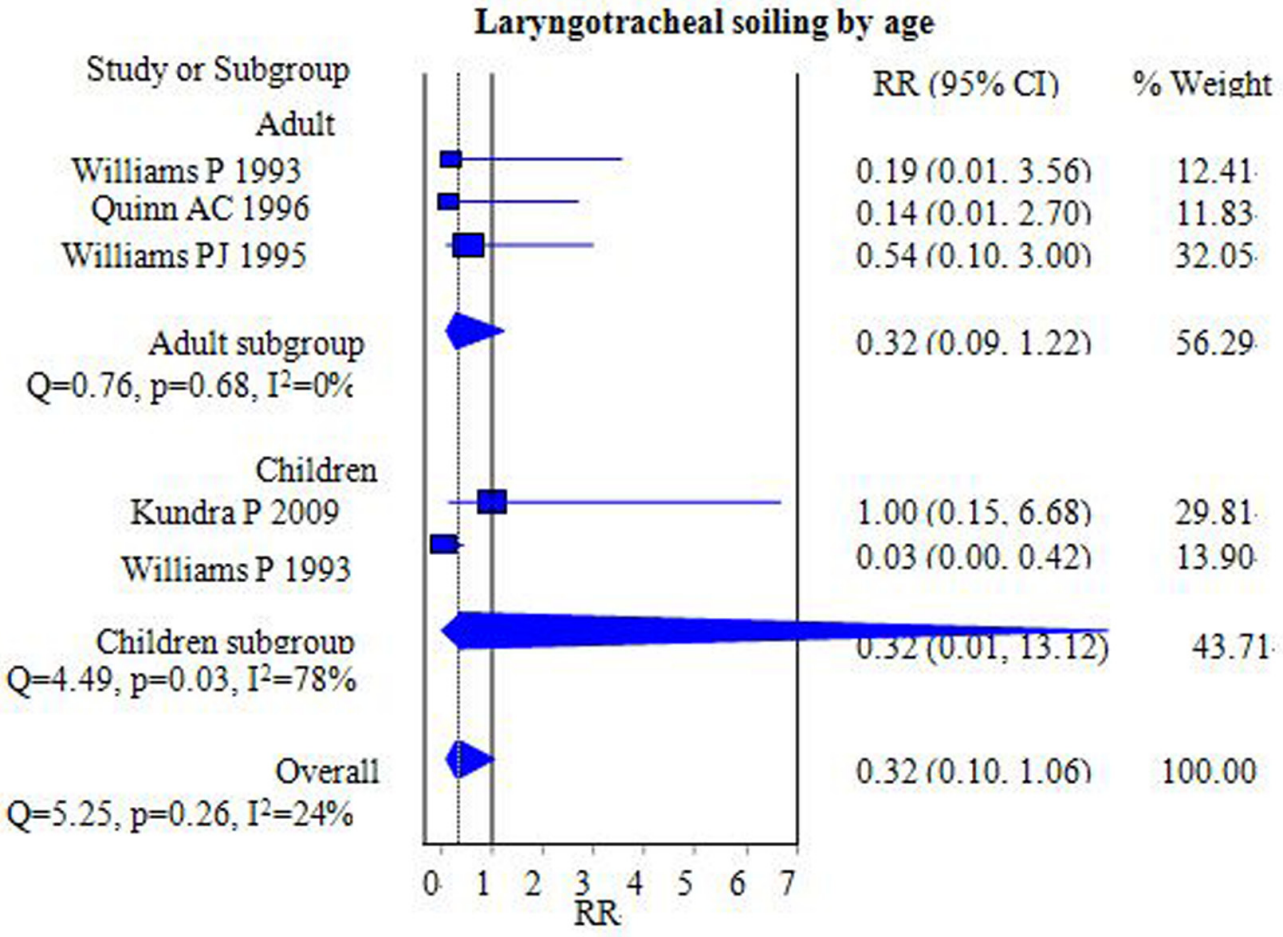
Forest plot demonstrating the incidence of laryngotracheal soiling: FLMA versus ETT. Subgroup analysis according to population age (adults versus children). The incidence was similar between the two groups.

Four studies that included 331 patients compared the incidence of laryngotracheal soiling [14,16,17,19]. The pooled results demonstrated no significant differences between the FLMA and ETT groups [RR = 0.32, 95% CI = (0.10–1.06); *I*^2^ = 24%] (Fig 8). Subgroup analysis by age group (adults versus children) showed no significant difference between the 2 groups [adults, RR = 0.32, 95% CI = (0.09–1.22); *I*^2^ = 0%; children, RR = 0.32, 95% CI = (0.01–13.12); *I*^2^ = 78%] (Fig 8).

#### Partial upper airway obstruction

**Fig 9:**
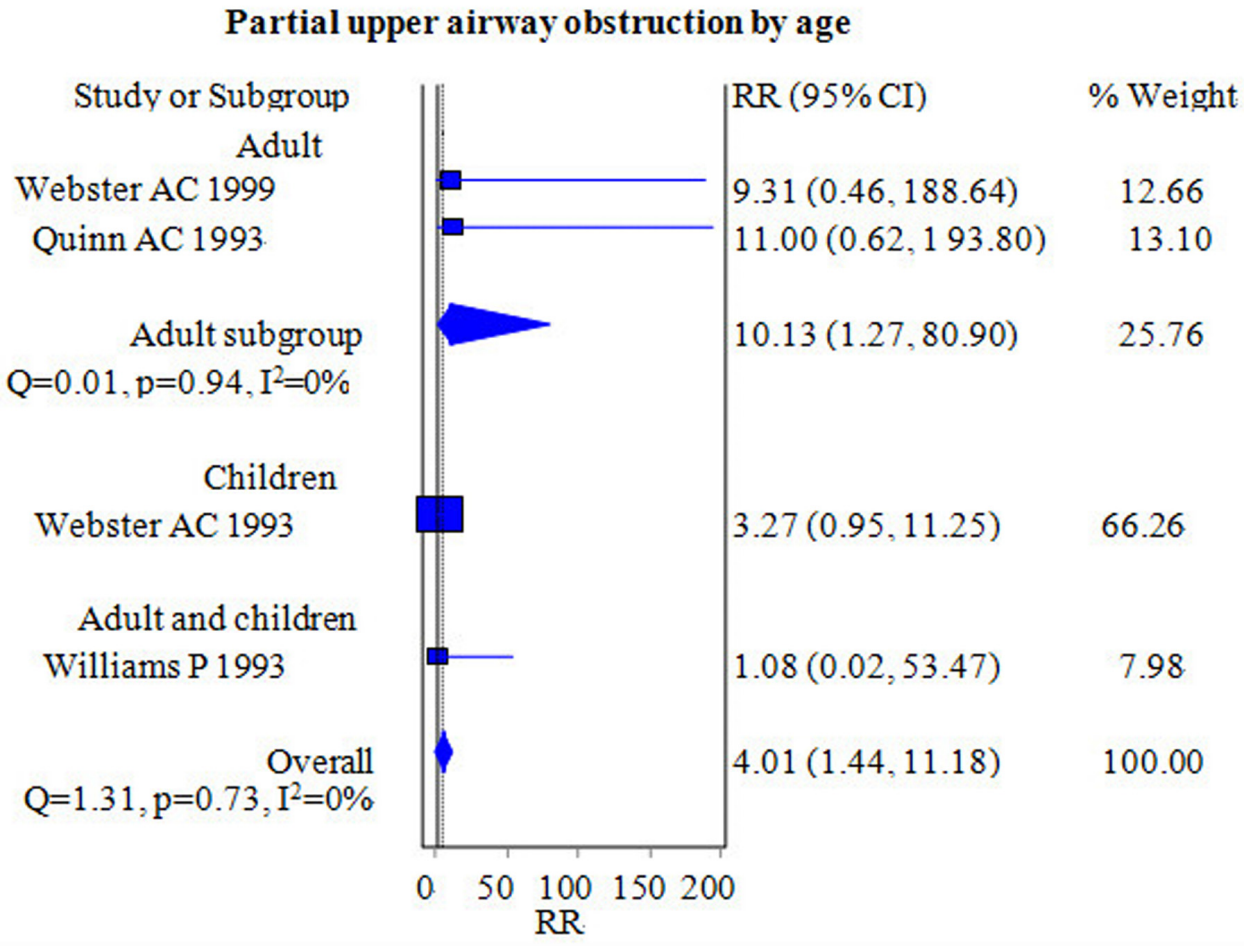
Forest plot demonstrating the incidence of partial upper airway obstruction. Subgroup analysis according to population age (adults versus children). The incidence in the FLMA group was significantly greater than that in the ETT group.

Four studies with 410 patients compared the incidence of partial upper airway obstruction during airway maintenance between the FLMA and ETT groups [6,16–18]. The incidence of partial upper airway obstruction in the FLMA group was significantly greater than that in the ETT group [RR = 4.01, 95% CI = (1.44–11.18); *I*^2^ = 0%] (Fig 9). The subgroup analyses indicated a greater incidence of partial upper airway obstruction in the FLMA group compared with the ETT group in the adult subgroup [RR = 10.13, 95% CI = (1.27–80.89); *I*^2^ = 0%] (Fig 9) but not in the pediatric subgroup [RR = 3.27, 95% CI = (0.95–11.25); *I*^2^ = 0%] (Fig 9).

#### Aspiration

**Fig 10:**
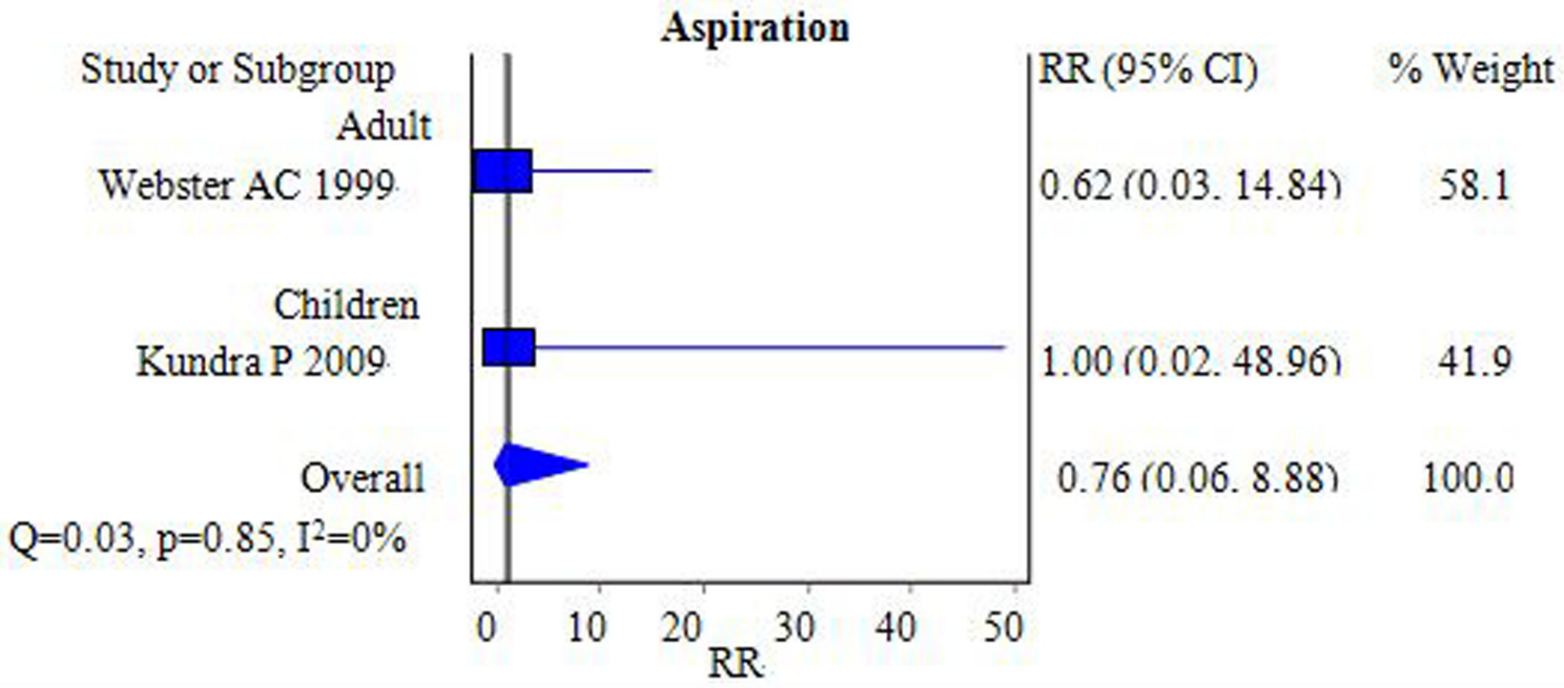
Forest plot demonstrating the incidence of aspiration. The incidence was similar between the two groups.

The incidence of aspiration was investigated in two studies [6,14]. By pooling the data, it was found that the incidence of aspiration was similar between the FLMA and ETT groups during anesthesia [RR = 0.76, 95% CI = (0.06–8.88); *I*^2^ = 0%] (Fig 10). In addition, the I^2^ was 0%, showing no heterogeneity.

#### Airway displacement

**Fig 11:**
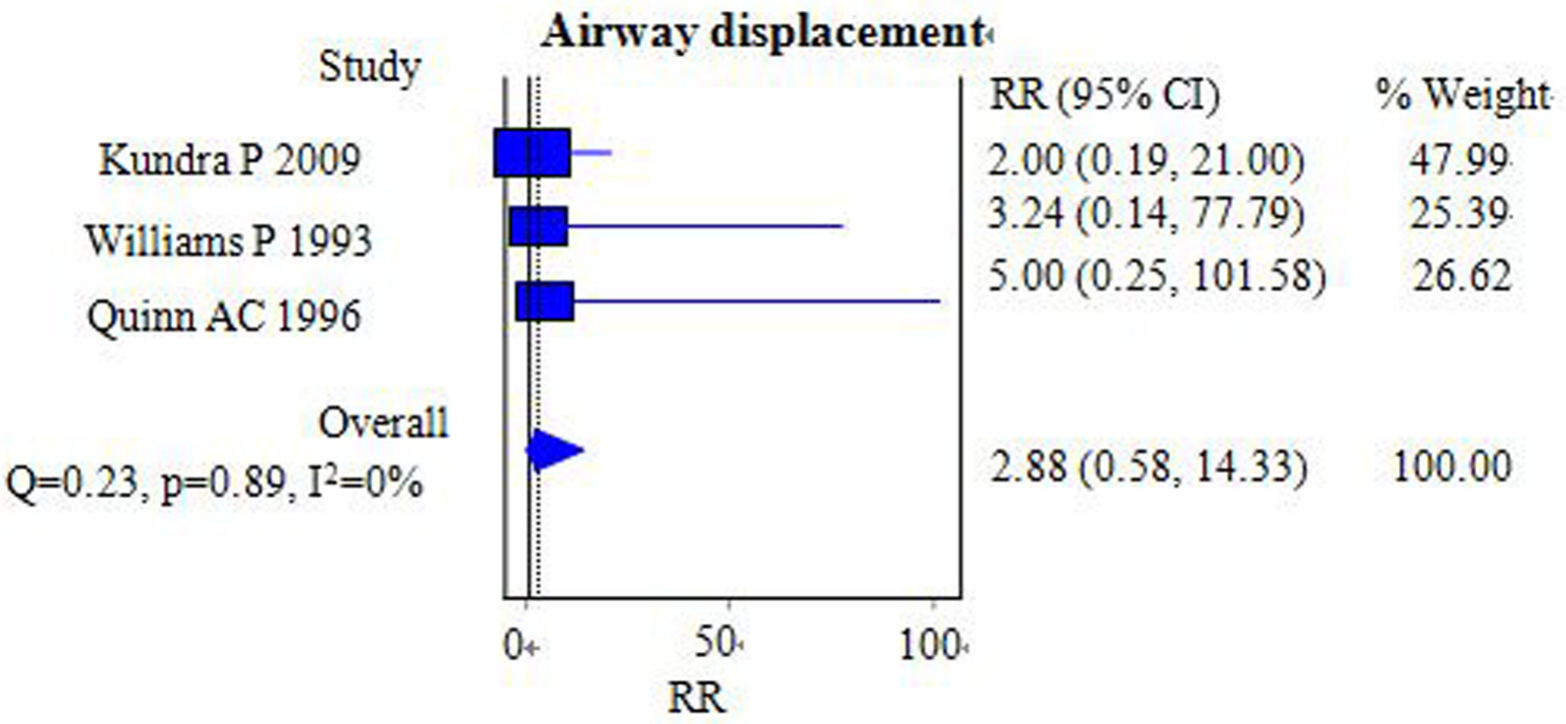
Forest plot demonstrating the incidence of airway displacement. The incidence was similar between the two groups.

Three studies including 266 patients compared the incidence of airway displacement between the FLMA and ETT groups [14,16,17]. The pooled results showed no significant differences between the two groups [RR = 2.88, 95% CI = (0.58–14.33); *I*^2^ = 0%] (Fig 11). Quinn AC et al reported that there were two cases in of airway displacement in the FLMA group and none in the ETT group. Omitting this study did not significantly change the overall pooled effect and 95% CI.

## Discussion

### Difficult intubation

In the current study, the incidence of difficulties with positioning the airway was similar between the FLMA and ETT groups. When the study by Webster AC was omitted, the overall pooled effect and 95% CI did not change significantly, indicating that the overall result was stable and robust. However, many studies have reported that the LMA is easier to insert than ETT. The possible explanation is the FLMA itself has a longer, narrower, non-rigid flexometallic tube that differs from that used with the LMA. The force cannot be transmitted along the shaft, which makes it more difficult to insert [20,21]. This variable incidence of difficulties can be explained by both differences in defining a difficult insertion and the variety of insertion techniques used by anesthesiologists with different levels of experience. The use of neuromuscular blocking drugs can help achieve adequate jaw relaxation and suppress upper airway reflexes, which also affects the incidence of experiencing difficulty when positioning the airway.

However, if a patient has a history of or indications for difficult intubation, a secure and definitive airway (ETT) should be obtained instead of the FLMA, which is not a secure airway and could be troublesome during surgery if problems occur, although there was no difference in the current analysis. However, the LMA is a great bridging tool when truly difficult intubation occurs. In almost all current difficult airway management guidelines, it is recommended that the LMA should be used after tracheal intubation has failed because of its ease of placement and its ability to provide oxygenation and ventilation effectively.

### Sore throat

Postoperative sore throat has been reported to be an inflammatory process because inflammatory mediators have been found in the tracheal mucosa after intubation [22]. Yu and Beirne [23] completed a meta-analysis in 2010 and reported that ETT had a greater incidence of sore throat (RR = 1.67, 95% CI = 1.33 to 2.11) compared with the LMA, but the difference was small, and the clinical significance of this difference is questionable. Our results show that the occurrences of sore throat at one hour and 24 hours postoperative were similar between the FLMA and ETT groups. This is consistent with Anthea Willam’s study [24]. A possible explanation is that the LMA cuff exerts pressure on the mucosa above the larynx [25], particularly when the FLMA is used. On the one hand, the lack of force that can be transmitted along the flexible tubing makes insertion difficult and seems to necessitate placing greater directional force on the tube to guide proper positioning [26]. On the other hand, mucosal pressure increases as a result of flexion forces from the tubing following taping [27]. Finally, there are differences in the use of nitrous oxide, the technique used for insertion, intracuff pressure and the surface material used in LMAs, and these factors may also impact the incidence of sore throat [28–30].

### Hoarseness

Hoarseness has been reported in as many as 50% of intubated patients and in approximately 15% of LMA patients [31]. Patient satisfaction may be decreased if hoarseness occurs. Our data suggest that a hoarse voice is more common in the ETT group than in the FLMA group, and this concurs with previous reports [23,32]. ETT may cause direct trauma to the vocal cords due to overextension of the neck during intubation or an overinflated cuff that keeps the vocal cords under constant pressure [33–35].

### Coughing and oxygen desaturation

The FLMA group had a significantly lower incidence of coughing and oxygen desaturation during the PACU recovery time compared to the ETT group. Our findings confirm the results of previous studies and systematic reviews [23,36]. Tracheal irritation by the endotracheal tube can cause serious and undesirable postoperative events. Coughing may lead to bleed at the surgical site, particularly in oral maxillofacial surgery and otorhinolaryngologic patients, and increases the risk of airway obstruction and suffocation. Intracranial or intraocular pressure can also be increased by coughing [37,38].

### Laryngospasms

Laryngospasms are one of the most common life-threatening respiratory adverse events. They have the potential to cause morbidity and mortality and may prolong hospitalization. Historically, the incidence of laryngospasms has been 0.78%-0.94% [39,40]. With refinements in anesthesia and surgical techniques, the rate of laryngospasms has decreased to 0.48%-0.1% [41,42]. In pediatric adenotonsillectomy, the incidence of laryngospasms ranges from 1.6% to 12.5% [13,43,44]. Identified risk factors for laryngospasms included younger age; upper respiratory tract infection; a history of at least two family members having asthma, atopy, or smoking; the presence of secretions; and a pre-existing airway anomaly. It remains controversial whether the use of the LMA is associated with a higher incidence of laryngospasms. Moreover, removal of the LMA early or after the return of airway reflexes is still debatable [45]. Our data have shown that there were no statistically significant differences between the FLMA and ETT groups in laryngospasms following the removal of the airway in either the adult or pediatric subgroups during adenotonsillectomy, which is the most common type of surgery. This finding is in close agreement with the findings of Dante Ranieri Junio et al [46]. However, some studies have found that the LMA has a higher risk of inducing laryngospasms than does ETT [41,47,48]. It has been speculated that this increased risk is caused by the use of the LMA or by accumulated secretions that may be a potent stimulus to the airway.

### Laryngotracheal soiling

Our results showed insignificant differences in the incidence of laryngotracheal soiling between the FLMA and ETT groups. In Pankaj Kundra’s study, laryngotracheal soiling was indicated by the presence of blood on the surface of the ETT inside the glottis and on the undersurface of the FLMA after removal [14], while the other three included studies used a fiberoptic bronchoscope to inspect the inside of the mask and the larynx or inserted it down the tracheal tube to view the trachea and detect blood soiling [16,17,19]. The incidence was lower in the FLMA group. P.J. Williams and A.C. Quinn et al found no laryngeal contamination and no blood on the laryngeal surface of the FLMA in any patient [16,17]. The FLMA appears to effectively protect the airway from soiling with blood and oropharyngeal secretions during surgery, particularly during ear, nose, and throat surgery [49,50].

### Aspiration

The LMA has disadvantages because it may be unable to protect the airway or be less efficient at protecting the airway from aspiration, and these are the leading factors that contribute directly to patient deaths caused by anesthesia according to the NAP4 summary. Our study showed that the incidence of aspiration was similar between the FLMA and ETT groups, but this result is unconvincing because only two subjects in the studies were analyzed, and the criteria and methods for diagnosing aspiration also differed. The FLMA should not be used in patients with a high risk of aspiration because patients treated with the LMA are vulnerable to different degrees of aspiration compared to ETT patients [51].

### Partial upper airway obstruction and airway displacement

We found that the incidence of partial upper airway obstruction and airway displacement in the ETT group was lower than that in the FLMA group, although the difference in airway displacement was not significant.

When the study by Quinn AC on airway displacement was omitted, the overall pooled effect and 95% CI did not change significantly, indicating that the result for airway displacement was stable and robust. It is generally considered that the application of a mouth gag, a defect in the palate, hyperextension of the head, and overinflation of the cuff can lead to airway obstruction and displacement [52]. Endotracheal tubes are much less likely to kink compared with the FLMA, and the experience of the operating surgeon and anesthesiologist may be the more powerful factor [53]. The sizes of the LMA tube and the mouth gag also contribute to the incidence of airway obstruction and displacement [54]. Because partial airway obstruction and displacement involving the FLMA is not always detected by clinical observation, we suggest that further prospective randomized trials should use fiberoptic bronchoscopy or ultrasound to help evaluate the position and ventilation of the LMA.

## Conclusion

In conclusion, in this meta-analysis, the FLMA had advantages over ETT in terms of lower incidences of hoarseness, coughing and oxygen desaturation. The data were insufficient to determine the differences in difficult intubation, laryngospasms, aspiration and postoperative sore throat. However, the incidence of partial upper airway obstruction was higher for the FLMA than for ETT. Although the incidence of airway displacement was lower and the incidence of laryngotracheal soiling was higher in the ETT group than in the FLMA group, these differences were not significant. Complications experienced with the use of the FLMA resulted in lower incidences of hoarseness, coughing and oxygen desaturation. Postoperative coughing and a hoarse voice are generally mild to moderate and can self-recover fairly quickly compared to airway obstruction and aspiration, which can potentially lead to more severe complications that may require extended care. Therefore, we conclude that in clinical practice, the anesthesiologist should fully consider the patient's physical condition when choosing between the FLMA and endotracheal tubes. It may also be problematic to use the FLMA in some populations, including morbidly obese patients, patients who require a mouth gag, and patients with a defect in the palate or who cannot remain in the prone position. In addition, the surgeon and anesthesiologist should be careful when applying the mouth gag, moving the head and neck, or performing oropharyngeal operations to avoid partial upper airway obstruction and airway displacement.

## Limitations

The present review has several limitations. First, very few studies were found to be eligible for inclusion, and therefore, it was not possible to classify every result into different age groups, such as children or adolescents, although we know there are some differences in the anatomy of the larynx between infants and adults. Second, the factors influencing airway complications in the perioperative period are multifactorial. They include improper endotracheal tube size, cuff design, lack of airway humidity, high anesthetic gas flow rates and adjacent tissues. Deficits in complete information such as this may affect the results. Third, an assessment of the severity of complications would be useful for clinical decision-making. However, the limited number of subjects in the studies did not allow for this type of analysis. Because of interstudy variations and the small size of the trials, further large-scale, multicenter studies are required to confirm or refute the results of this meta-analysis.

## Supporting Information

S1 Table PRISMA Checklist. PRISMA Checklist for this meta-analysis.
(DOC)
